# Progress in Ophthalmology

**Published:** 1898-05-07

**Authors:** 


### Progress in Ophthalmology.

(*Continued from page* 47.)

**Iritis After Slight Corneal Injuries.**—Antonelli[1] draws attention to this form of iritis and says, "This complication, which gives rise to considerable pain and entails a greater or longer disability for work, presents itself in the form of an iritis slight in intensity, but which never lasts less than three or four weeks, the pericorneal injection persisting the whole of that time, notwithstanding that the original corneal wound may have completely healed up, with disappearance of all pain." The form of iritis he describes as being intermediate between the serous and plastic. The pupil is easily dilatable if atropin is used early, and its effect kept up by instillations daily. Adhesions rarely form nor are there any exudative deposits on back of cornea or increase of tension. The anterior chamber becomes slightly deeper and the aqueous muddy. This iritis is not a regular septic one, but he prefers to call it toxic and compares it to that described by Hilbert, where iritis accompanies injuries, chiefly chemical ones, of the anterior segment of the eyeball without any actual wound of the cornea properly so-called. He says that this form of iritis is most often seen after small punctured wounds of the cornea such as would be made by the sharp point of a palm or other leaf, and lays down, first, that in all such cases the prognosis must be guarded, for even if the corneal wound heals up rapidly, the subsequent iritis may prolong the trouble for five or six weeks; and second, that all such wounds of the cornea must be treated with atropin if produced by a foreign body, especially if the edges of the wound look grey or have that metallic rusty tinge which is seen when they are caused by chips of iron or steel.

**Etiology of Dacryocystitis.**—J. W. H. Eyre, who studied this disease in the Bacteriological Laboratory of Guy's Hospital,[2] comes to the conclusion that dacryocystitis is not always due to stricture of the lachrymal ducts. He is willing to admit the presence of stricture in some cases, and says that it is no doubt a predisposing cause, but will not admit that in all, or, indeed, in many of the cases, stricture is the cause, inasmuch as it is absent. He believes the cause to be invasion of the conjunctival sac with streptococcus pyogenes longus, causing an acute conjunctivitis. Owing to the extra lachrymal secretion the streptococcus is washed into the lachrymal sac, then an excessive secretion of mucus and leucocytes into the sac in the endeavour to remove the streptococci, and finally invasion of the mucous membrane lining the interior of the sac by the streptococcus, which eventually gets into the submucosa and cellular tissue surrounding the sac, and the consequent formation of pus in those situations to which it has gained access. The same author,[3] in writing of the study of the bacteriology of the conjunctival sac, made cultures from 150 eyes, in 75 of which he found no colonies of bacteria. In those which did yield bacteria, he found 28 different forms of micro-organisms, and concludes from his observations that the conjunctival sac frequently contains micro-organisms which may or may not be pathogenic. It may be sterile at the particular moment when an observation is made. The upper formix is much oftener sterile than the lower. The sterility of the conjunctival sac is due to the mechanical flushing of its mucous surface by the lachrymal secretion, aided, perhaps, by the bactericidal character of the fluid.

**The Physiological Role of the Leucocytes in Corneal Wounds.**—M. L. Ranvier[4] says: In observing a cornea that has been cut, and in which the wound is being filled with epithelial cells, there are three facts among those noticed worthy of attention—(1) The epithelium which occupies the surface of a wound presents the appearance of a very active cellular multiplication; indeed, a certain number of nuclei are observed which show the different forms of multiplication by indirect division. (2) Large numbers of the epithelial cells have penetrated into the gaps or abrasions of the surface resulting from the tearing up of the superficial corneal layers. These cells form shoots variously entangled, due to their being massed together, which recall what is observed in cancers. (3) The central portion of the wound—that which is not yet covered with epithelium—presents a considerable number of lymphatic cells. One would believe he saw at this level a small purulent nodule in the stroma of the cornea. The lymphatic cells are far less numerous in those parts that have been covered again by epithelium, though this epithelium has been replaced so short a time. It seems as if the cells have been driven back and made to accumulate in the denuded region. The true explanation of these facts is, however, that the lymphatic cells are attracted by the air. Furthermore, he states: If we now examine the lymphatic cells accumulated in this little region of the corneal stroma that is denuded of its epithelium, we observe that most of those cells are clear, rounded, limited by a double contour, and contain several small nuclei; these are pus corpuscles. Formerly these corpuscles were considered as dead lymphatic cells or cells that were in process of destruction. Since then investigators have wished to make a special form of leucocyte of the polynuclear variety, but the author does not believe in this classification, as he has seen the mononuclear leucocyte become transformed under his eyes into the polynuclear variety. He also believes he can add that these modified cells have given up to the tissue with which they are in contact a part of the nutritive substances with which they were loaded. In the simplest wounds of the cornea, such as a simple incision, the migratory cells soon come to take part in the reparative process. A certain number of them are seen on the lips of the wound at the end of four hours. Twenty hours later there are still a few of them present. At this time the solution of continuity is filled by epithelial cells, which present all the signs of nutritive hyperactivity, They are large, filled with juice, and have large nucleæ. They cannot accomplish this work without being abundantly nourished. There are no blood vessels to bring them their nourishment, and so the simplest way to account for this nourishment is by attributing this function to the migratory cells. On the edges of a wound these cells always preserve the same characters, they have lost their protoplasmic chromatin, and they have multiple nuclei; often their protoplasm having been entirely dissolved their nuclei are set free. The migratory cells in passing into the interior of the tissue can then give up to them a part of the substances which they enclose, or it may happen even that their entire protoplasm is dissolved, and that the materials of which it is formed are dissipated in the nutritive plasma on which the organs live. These corpuscles enter all parts of the body where the blood vessels cannot or do not penetrate, as, for instance, the cornea.

**Hyoscin and Hyoscamin as Mydriatics.**—Emmert[5] says he has for the last sixteen years found hyoscin to be one of the most constant and reliable mydriatics we have, and that this has been the opinion of many others. As the hydrobromate, it is a very stable salt, and has been introduced into both the Swiss and German pharmacopœias under the name of scopolomin, which is identical with hyoscin. Its advantages over atropin he states to be: (1) It dilates the pupil more quickly; (2) the mydriasis doe s not last so long, and the paralysis of accommodation lasts about the same time, and this is an advantage in healthy eyes; (3) it cures inflammatory affections of the eye quite as quickly as atropin; (4) even in prolonged use it has no local irritant action such as atropin conjunctivitis; (5) it is five times more powerful; (6) when used in solutions as strong as 1-500 or 1-350, any general intoxication is very rare, and even if present quickly passes off, and is not dangerous—a solution of 1-1,000 is usually sufficient, and never causes poisoning; (7) intra-ocular tension is not influenced in chronic glaucoma, but in acute glaucoma its use is contra-indicated; (8) instead of stimulating the cerebral cortex and quickening the pulse like atropin, it has a paralysing action on the former, and slows the pulse, and hence is much safer in heart disease. Considering the weak solutions necessary, it is practically as cheap as atropin. As regards hyoscamin, its infrequent use as a mydriatic is not due to an uncertain action, but because it has no advantage over atropin.

[1] Med. Chronicle, Feb., 1898. [2] Amer. Journ. Med. Science, Mar., 1898. [3] Amer. Journ. Med. Science, Jan., 1898. [4] Amer. Med. Surg. Bulletin, Feb. 10. 1898. [5] Epit. Brit. Med. Journal, Mar. 5. 1898.

